# A statistical model for forecasting probabilistic epidemic bands for dengue cases in Brazil

**DOI:** 10.1016/j.idm.2025.07.014

**Published:** 2025-08-05

**Authors:** Laís Picinini Freitas, Danielle Andreza da Cruz Ferreira, Raquel Martins Lana, Daniel Cardoso Portela Câmara, Tatiana P. Portella, Marilia Sá Carvalho, Ayrton Sena Gouveia, Iasmim Ferreira de Almeida, Eduardo Correa Araujo, Luã Bida Vacaro, Fabiana Ganem, Oswaldo Gonçalves Cruz, Flávio Codeço Coelho, Claudia Torres Codeço, Luiz Max Carvalho, Leonardo Soares Bastos

**Affiliations:** a Scientific Computing Program, Oswaldo Cruz Foundation, Rio de Janeiro, RJ, Brazil; b Federal University of Minas Gerais, Belo Horizonte, MG, Brazil; c Barcelona Supercomputing Center (BSC), Barcelona, Spain; d Graduate Program in Epidemiology in Public Health, Sergio Arouca National School of Public Health, Oswaldo Cruz Foundation, Rio de Janeiro, RJ, Brazil; e School of Applied Mathematics, Getulio Vargas Foundation, Rio de Janeiro, RJ, Brazil; f Institute of Pure and Applied Mathematics and Technology (IMPA Tech), Rio de Janeiro, RJ, Brazil

**Keywords:** Dengue, Forecasting model, Vector-borne diseases, Disease surveillance, Epidemic monitoring

## Abstract

Dengue is a vector-borne disease and a major public health concern in Brazil. Its continuing and rising burden has led the Brazilian Ministry of Health to request for modelling efforts to aid in the preparedness and response to the disease. In this context, we propose a Bayesian forecasting model based on historical data to predict the number of cases 52 weeks ahead for the 118 health districts of Brazil. We leverage the predictions to build probabilistic epidemics bands to be used for dengue monitoring. We define four disjoint probabilistic bands (≤50% (50%, 75%] (75%, 90%], and >90%), based on the percentiles of the predicted cases distribution and interpreted according to the historical number of cases and past occurrence probability (below the median, typical; moderately high, fairly typical; fairly high, atypical; exceptionally high, very atypical). We performed out-of-sample validation for 2022–2023 and 2023–2024 and forecasted 2024–2025. In the 2022–2023 and 2023–2024 seasons, the epidemic bands followed the observed cases’ curve shape, with a sharp increase after January and a decline after the peak around April. In 2022–2023, the observed number of cases (1,436,034) was slightly above the estimated 75% percentile (1,405,191), being classified as “fairly high, atypical”. Most health districts in South Brazil showed exceptionally high numbers of cases during this season. The situation worsened in 2023–2024 and the observed number of cases (6,454,020) was way above the 90% percentile (2,221,557), characterising an “exceptionally high, very atypical” season. For the 2024–2025 season, we estimated a median number of cases of 1,526,523 (maximum value for the “below the median, typical” probabilistic epidemic band. The maximum estimated values for the upper bands were 2,213,282 (moderately high, fairly typical) and 3,803,898 (fairly high, atypical) with the upper limits of the probabilistic epidemic bands of 1,452,359. Probabilistic epidemic bands serve as a valuable monitoring tool by enabling prospective comparisons between observed case curves and historical epidemic patterns, facilitating the assessment of ongoing outbreaks about past occurrences.

## 1 Introduction

Dengue is a vector-borne disease on the rise globally caused by four different viral serotypes and transmitted to humans by *Aedes aegypti* and *Aedes albopictus* mosquitoes. In the Americas only, dengue affected around 13 million people and caused more than 8 thousand deaths in 2024, exceeding the region's historical annual record (PAHO, 2025; Lenharo, 2023). In the same year, Brazil faced its most severe dengue epidemic since the introduction of the DENV-1 and DENV-4 serotypes in Roraima in 1981 (Osanai et al., 1983). Over 6.6 million cases and 6 thousand related deaths were reported (Brasil, Ministério da Saúde, 2025a). This dire situation has motivated the Brazilian Ministry of Health to reach out for modelling approaches that could guide interventions in a timely manner.

Forecasting models can be useful for enhancing public health preparedness by anticipating potential epidemic outbreaks and informing resources allocation and timely interventions, especially when they provide estimates in high spatial and temporal resolutions. Specifically for dengue, forecasting models have been proposed using statistical methodologies with different levels of complexity (Colón-González et al., 2021b, Colón-González et al., 2021a; Karasinghe et al., 2024; Pirani et al., 2024) and, in recent years, machine learning methods (Stolerman et al., 2019; Al Mobin, 2024; Sebastianelli et al., 2024). With different methodologies presenting interesting results for both explaining and predicting dengue outbreaks, an important question remains on what methods can be accurate while being useful and operationally viable for health authorities.

In this paper, we propose a Bayesian spatio-temporal forecasting model based on historical surveillance data to predict dengue cases up to 52 weeks in the future for all 118 health districts of Brazil. These predictions correspond to what would be expected under similar conditions as those observed in the past. Therefore, they can be used for monitoring to detect atypical scenarios. To develop this monitoring tool, we build probabilistic epidemic bands based on percentiles of the predictive cases distributions and interpret them considering the historical median and the probability of past occurrence.

## 2 Methods

### 2.1 Study context and data

A health district is an administrative grouping of neighbouring municipalities from the same Federal Unit (state) that share socioeconomic and transport infrastructure (Supplementary Figure A1). Their purpose in the Brazilian Unified Health System (*Sistema Único de Saúde* - SUS) is to organise the health care network in an integrated manner to correct access inequalities.

We used surveillance data from the Notifiable Diseases Information System (*Sistema de Informação de Agravos de Notificação* - SINAN) from the Brazilian Ministry of Health, where dengue is a disease of mandatory reporting. A reported case of dengue corresponds to a patient who sought medical care and received a suspected or confirmed diagnosis of dengue upon medical evaluation. Later, a reported case can be discarded for reasons such as confirmation of another aetiological agent.

Anonymised dengue case data are openly available at the individual level from https://datasus.saude.gov.br/transferencia-de-arquivos/. Cases missing information on the municipality of residence were excluded. The municipality of residence was used to identify the health district of residence of cases. Most of dengue cases occur in summer and fall, so we opt to start a season in spring, on epidemiological week 41, and ending a season on week 40 of the following year. Then, cases were grouped by health district of residence, season and season week of symptoms onset. We included notified cases of dengue symptoms onset between October 11, 2015, and October 05, 2024, and excluded all discarded cases, obtaining a dataset of “probable cases” (Supplementary Figure A2).

### 2.2 Statistical model

We propose a Bayesian negative binomial model with structured random effects for season week and unstructured random effects for the season for each health district. The forecast was performed retrospectively for the seasons 2022–2023 and 2023–2024 and prospectively for season 2024–2025. Predictions of the number of dengue cases are obtained from samples of an approximate posterior predictive distribution per week and health district. These predictions can be further aggregated to lower spatial and temporal resolutions (e.g. Federal Units and season).

#### 2.2.1 Model specification

Let *Y*_*t*,*h*_ be the number of dengue cases at time *t* and health district *h*. We then assume that

(1) $$Y_{t,h}∼\mathrm{NegBin}\left(\lambda_{t,h},\phi_{h}\right),$$

where *ϕ*_*h*_ > 0 is an overdispersion parameter and *λ*_*t*,*h*_ is the expected number of dengue cases at time *t, t =* 1, 2, …, *T*, in health district *h*, *h* = 1, 2, …, *H,* modelled as

(2) $$\log\left(\lambda_{t,h}\right)=\alpha_{h}+\beta_{w\left[t\right],h}+\gamma_{s\left[t\right],h},$$

where *α*_*h*_ are health district-specific intercepts, *β*_*w*[*t*],*h*_ are cyclic second-order random walk effects by week *w*, *w* = 1, 2, …, 52, and health district *h*, and *γ*_*s*[*t*],*h*_ independent Gaussian random effects for season *s*, *s* = 1, 2, …, *S*, and health district *h*. The cyclic term on the week random effects means that the first week, week 1, is correlated with the last one, week 52.

The model specification is completed by setting penalized complexity (PC) priors for the remaining parameters (Simpson et al., 2017). For the overdispersion parameter *ϕ*_*h*_ a PC Gamma is chosen with a limiting Poisson case as a baseline. For the random effects variances $\sigma_{\beta }^{2}$ and $\sigma_{\gamma }^{2}$, it was assumed the same PC prior for the precision with parameters (3,0.01) such that *P*(*σ* > 3) = 0.01, where the standard deviation *σ* = *τ*^1/2^.

The posterior predictive distribution of the cases of the following year, *s* = *S* + 1, for each health district, *h*, and all 52 weeks is given by

(3) $$\begin{array}{ll} & p\left(Y_{T+1,h},Y_{T+2,h},\ldots ,Y_{T+52,h}|y_{1,h},y_{2,h},\ldots ,y_{T,h}\right)=\\ & \int_{\theta_{h}\in \Theta_{h}}p\left(Y_{T+1,h},Y_{T+2,h},\ldots ,Y_{T+52,h}|\theta_{h}\right)p\left(\theta |y_{1,h},y_{2,h},\ldots ,y_{T,h}\right)d\theta_{h} \end{array}$$

where $\theta_{h}=\left[\left\{\alpha_{t,h}\right\},\left\{\beta_{w,h}\right\},\left\{\gamma_{s,h}\right\},\phi_{h},\sigma_{\beta }^{2},\sigma_{\gamma }^{2}\right]$, the first term inside the integral is the likelihood (1) evaluated at the new data and the second is the posterior distribution of *θ*_*h*_. Inference is done via integrated nested Laplace approximation (Martins et al., 2013; Rue et al., 2009), and samples from an approximation for the posterior predictive distribution (3) using the Gaussian latent parameters and the discrete version of the hyperparameters distribution can be obtained using the inla.posterior.sample() function implemented in the INLA package.

#### 2.2.2 Probabilistic epidemic bands and classification

From the samples, we obtain the 50th, 75th, and 90th percentiles of the predictive distribution of the cases for each health district, *h*, and week, *w*. These percentiles establish probabilistic epidemic thresholds, delineating four distinct probability bands of ≤50%, (50%, 75%], (75%, 90%], and >90%. We classify these probabilistic epidemic bands based on the historical case distribution and past occurrence probabilities, designating them as follows: below the median, typical; moderately high, fairly typical; fairly high, atypical; and exceptionally high, very atypical (Table 1). The historical median is defined as the 50th percentile of the predicted case distribution from 2015 up to the season immediately preceding the one being forecasted.

**Table 1:**
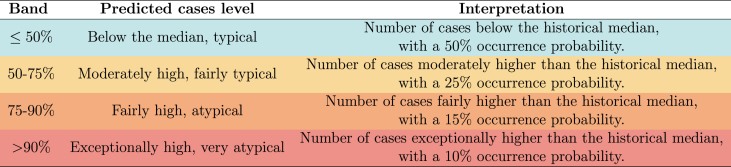
Proposed classification of predicted cases level with the interpretation.

#### 2.2.3 Model validation

We performed out-of-sample validation for two seasons. For forecasting the 2022–2023 season, we used data by epidemiological week 40/2022, while for forecasting 2023–2024 we modelled data by epidemiological week 40/2023. We visually compared the predictive posterior probabilistic epidemic bands with the observed values of number of cases by season week at the national and Federal Unit levels. For each season, we classified and depicted the health districts according to the epidemic band within which the observed number of dengue cases fell.

Analyses were performed in R (version 4.4.1) (R Core Team, 2024) using R-INLA (version 24.06.27) (Martins et al., 2013; Rue et al., 2009). All data and code are available at https://github.com/AlertaDengue/baseline_paper/.

## 3 Results

In this section, we first present the results of the retrospective forecasting for two seasons (2022–2023 and 2023–2024) and compare them with the observed number of dengue cases. Next, we show the results of the prospective forecasting for the 2024–2025 season. An interactive dashboard was developed to accompany this publication, allowing users to explore all results in greater detail across different spatial resolutions, including the health district level: https://dcpcamara-picinini-freitas-et-al-2025.share.connect.posit.cloud/.

### 3.1 Retrospective forecasting and model validation

For the 2022–2023 season, the observed number of dengue cases (1,436,034) was slightly above the estimated 75% percentile (1,405,191), falling within the 75–90% probabilistic epidemic band and classified as “fairly high, atypical” - with a low probability of occurrence considering the historical data (Table 2). The 2023–2024 season was very atypical, with an unprecedented number of observed cases of over 6.4 million. This number vastly exceeded the 90% percentile of the number of predicted cases, with a very low probability of occurrence.

**Table 2:** Percentiles of the predicted number of dengue cases by season and number of observed cases, 2022–2025, Brazil.

		2022–2023	2023–2024	2024–2025
**Percentile**	50%	980,013	1,070,672	1,526,523
	75%	1,405,191	1,452,359	2,213,282
	90%	2,371,785	2,221,557	3,803,898
**Observed**		1,436,034	6,454,020	

**Fig. 1:**
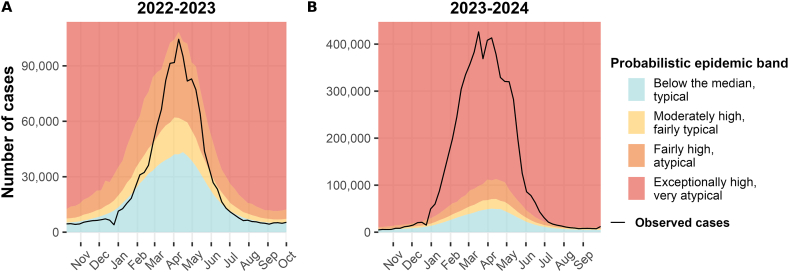
Estimated probabilistic epidemic bands compared with the observed number of dengue cases by week, 2022–2023 (A) and 2023–2024 (B) seasons, Brazil.

In Fig. 1, the national-level weekly estimations are depicted against the curve of observed dengue cases for the 2022–2023 (panel A) and 2023–2024 (panel B) seasons. The colours depict the probabilistic epidemic bands, which indicate the predicted case level as proposed in Table 1. In both panels of Fig. 1, we observe that the epidemic band shapes follow the curve of the observed cases, with a sharp increase after January and a decline after the peak around April.

In 2022–2023 (Fig. 1A), the observed cases curve fell within the “below the median, typical” band (in cyan) at the beginning of the season, quickly increasing and reaching the “moderately high, fairly typical” (in yellow) and “fairly high, atypical” (in orange) after February. Only during the peak (season week 27, in April), the observed number of cases was “exceptionally high, very atypical” (in red).

A markedly different scenario emerged in the 2023–2024 season, during which the observed dengue case curve remained within the “exceptionally high, very atypical” band for most of the season (Fig. 1B). This band classification represents a highly unlikely occurrence based on historical data.

**Fig. 2:**
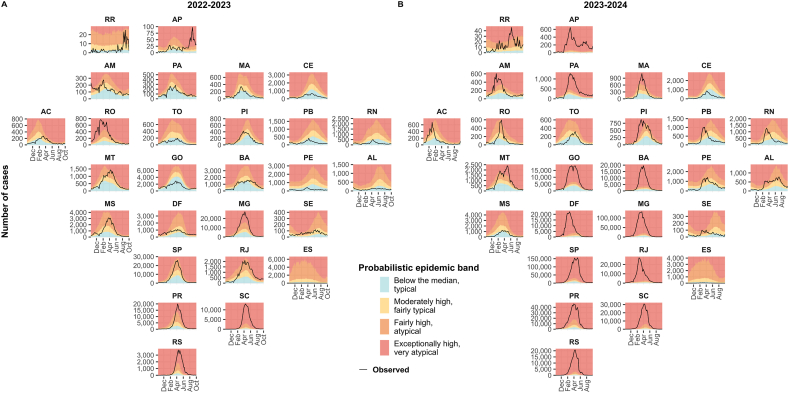
Estimated probabilistic epidemic bands compared with the observed number of dengue cases by week and Federal Unit in 2022–2023 (A) and 2023–2024 (B) seasons.

The probabilistic epidemic bands by week for each Federal Unit against the observed cases curve is depicted in Fig. 2 for seasons 2022–2023 and 2023–2024.

Many Federal Units had fewer observed cases than the historical median in 2022–2023 (Fig. 2A), especially in Northeast Brazil (Maranhão - MA, Ceará - CE, Rio Grande do Norte - RN, Paraíba - PB, Pernambuco - PE, and Alagoas - AL). On the other hand, all southern Federal Units (Paraná - PR, Santa Catarina - SC, and Rio Grande do Sul - RS) had an exceptionally high number of dengue cases. This very atypical situation in the South continued during the 2023–2024 season (Fig. 2B) when other Federal Units from all Brazilian regions also had an exceptionally high number of observed cases (North: Amapá - AP and Pará - PA; Mid-West: Goiás - GO and Federal District - DF; Northeast: Bahia - BA; Southeast: Minas Gerais - MG, São Paulo - SP, and Rio de Janeiro - RJ). Interestingly, for the 2023–2024 season, the curve of observed dengue cases in Paraíba (PB), Rio Grande do Norte (RN), Pernambuco (PE) and Alagoas (AL) peaked earlier than predicted considering the historical data.

**Fig. 3:**
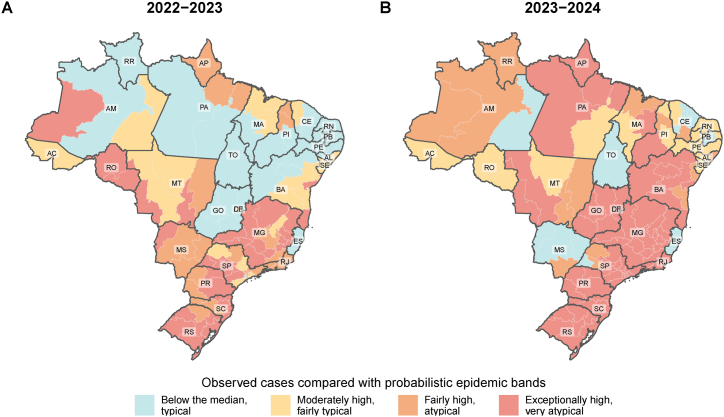
Observed number of cases compared with probabilistic epidemic bands by health district in the 2022–2023 (A) and 2023–2024 (B) seasons.

The maps in Fig. 3 illustrate the probabilistic epidemic band in which the total observed dengue cases for seasons 2022–2023 (panel A) and 2023–2024 (panel B) fell within across different health districts. By increasing the spatial resolution, we can clearly identify heterogeneity within Federal Units. For instance, during the 2022–2023 season, one health district in Amazonas (AM) experienced an exceptionally high number of dengue cases, while the rest of the state recorded case counts below the historical median. Although some health districts reported exceptionally high case numbers in 2022–2023 (30 out of 118), the majority had either fewer (45 out of 118) or moderately higher (27 out of 118) cases than the historical median (Fig. 3A). In contrast, during the 2023–2024 season (Fig. 3B), most health districts (70/118, or 59.3%) experienced an exceptionally high number of dengue cases, underscoring the unprecedented scale and severity of this epidemic.

### 3.2 Prospective forecasting for the 2024–2025 season

For the 2024–2025 season, the estimated upper limits for the probabilistic epidemic bands were 1,526,523 (50%), 2,213,282 (75%) and 3,803,898 (90%) dengue cases in Brazil (Table 2). These represent a substantial increase in the predicted number of dengue cases compared to the estimates for the previous two seasons, influenced mainly by including the 2023–2024 season data in the model.

**Fig. 4:**
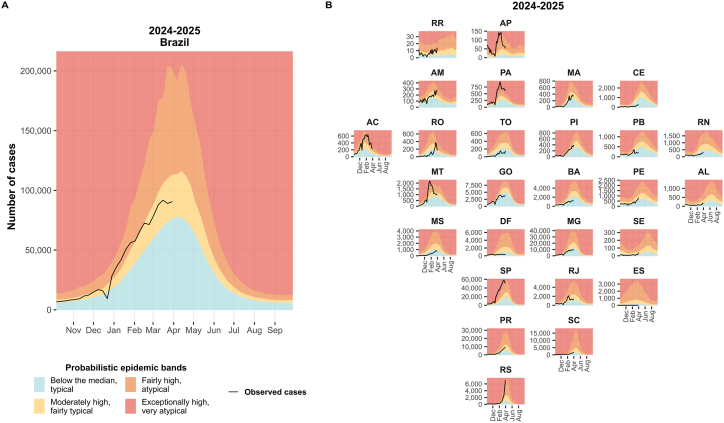
Forecasted probabilistic epidemic bands of weekly dengue cases for the 2024–2025 season in Brazil (A) and by Federal Unit (B).

Fig. 4A also shows the increase in the thresholds of the forecasted probabilistic bands of dengue cases for the 2024–2025 season compared to the previous two seasons. Fig. 4B depicts the forecasted probabilistic epidemic bands for each Federal Unit. An increase in the thresholds is evident for MG, SP, PR, SC and RS, as well as for GO, RJ and BA. These Federal Units experienced an exceptionally high number of cases in 2023–2024.

Supplementary Figures A3–A29 provide enlarged versions of the plots from Fig. 2, Fig. 4B, offering a clearer visualization of the probabilistic epidemic bands by Federal Unit over the three seasons.

## 4 Discussion

We successfully estimated probabilistic epidemic bands for dengue in Brazil using a forecasting model with only case number data. The predictions, derived from historical data, provide a baseline of what is expected for the next dengue season, with uncertainty, under conditions similar to those in the past. Due to this characteristic, our model predictions are helpful for prospective outbreak monitoring. To facilitate its use as such, we propose a classification of probabilistic epidemic bands that facilitates the visualization and interpretation of an ongoing dengue situation.

The out-of-sample validation demonstrated that our model generates reliable predictions for more typical dengue seasons. However, its accuracy declines in regions experiencing recent or ongoing dengue emergence (e.g., southern Brazil) or during seasons that deviate significantly from historical patterns (e.g., the 2023–2024 season). Since the model relies exclusively on historical case data, it cannot anticipate epidemic scenarios that have never occurred in the past. The 2023–2024 epidemic was unprecedented, more than 3 times bigger than the second-largest dengue epidemic in Brazil in 2018–2019 (Supplementary Figure A2). This highly atypical season was likely the result of a combination of dengue expansion and intensified transmission dynamics. Dengue has been expanding geographically to new areas, putting previously unaffected populations at risk, a phenomenon that has intensified in recent years, likely due to global warming (Codeco et al., 2022; Barcellos et al., 2024). In addition, changes in meteorological conditions promoted by the El Niño Southern Oscillation (ENSO) climatic event may have favoured the biology of the vector *Ae. aegypti* and boosted both dengue expansion and transmission (Pirani et al., 2024; Vincenti-Gonzalez et al., 2018).

Therefore, climate, population immunity, and serotype circulation information could enhance our model when aiming at accurate and precise predictions. Data on population immunity and serotype circulation are often very limited. On the other hand, climate data have been increasingly made available and used to model vector-borne diseases (de Sousa et al., 2018; Colón-González et al., 2013, 2021b, 2023, 2021a; Sophia et al., 2025; Lowe et al., 2015, 2017; Chou-Chen et al., 2023; Leung et al., 2023). We found that dengue outbreaks in Northeast Brazil occurred sooner than expected in 2023–2024. Suppose this was a consequence of changes in temperature or precipitation patterns. In that case, including climate data could have predicted the anticipation of the epidemic curve in the short term. In the long term, however, to estimate future climate data would be necessary. Additionally, the model would have to predict changes in climate patterns, which is not a trivial task. To address this challenge, long-term predictions with climate data may be built for different climatic scenarios (Colón-González et al., 2013, 2021a, 2023; Sophia et al., 2025). This, however, may compromise the usability of the predictions by the public health system. Such more complex structures considering climate factors, which may or may not have lagged and non-linear effects (Lowe et al., 2018, 2021; Ortega-Lenis et al., 2024), can be explored in future work and by other research groups using our model as a “baseline model”.

It is straightforward to devise a monitoring tool to detect early shifts if a dengue situation differs from historical patterns with the probabilistic epidemic bands to generate alerts in a timely manner. This feature is further strengthened in combination with nowcasting models, which provide estimates of case numbers corrected for reporting delays in disease surveillance data (Bastos et al., 2019; Günther et al., 2021; McGough et al., 2020; Stoner & Economou, 2020). These procedures further align with the objectives of the “Action Plan for Reducing Dengue and other Arboviruses” from the Brazilian Ministry of Health (Brasil, Ministério da Saúde, 2024). In the “National Contingency Plan for Dengue, Chikungunya and Zika”, the Ministry of Health suggests the use of control charts based on quartiles and medians of past distributions for risk assessment (Brasil, Ministério da Saúde, 2025b). In this regard, applying our model instead represents a similar idea with the same goal but an improvement for epidemic monitoring due to its statistical robustness. When comparing the probabilistic epidemic bands with the control charts recommended by the Brazilian Ministry of Health, the latter tended to produce more frequent warnings, including during low-incidence periods or shifted seasonal peaks, whereas our model issued fewer but apparently more accurate warnings, better aligned with substantial deviations. Details of this comparative analysis are provided in Supplementary Appendix B.

To operationalize the model for early warning, real-time data availability is essential, ideally complemented by nowcasting models, as mentioned. To support early warning, our model provides probabilistic forecasts grouped into four bands that, rather than issuing binary alerts, can serve as input for response strategies according to the risk level. This proposal aligns with the four operational stages defined in the Ministry of Health's contingency plan: normality (below the historical median), mobilisation (between the median and the third quartile), alert (above the third quartile), and epidemic (exponential increase above the third quartile). In this context, an increase of cases into the “moderately high, fairly typical” band could suggest mobilisation; crossing into the “fairly high, atypical” band would issue an alert; and reaching the “exceptionally high, very atypical” band may indicate an epidemic is going on. However, we emphasize that this is a proposed framework, and that defining operational thresholds and issuing alerts must be done in coordination with the responsible public health authorities, such as the Ministry of Health, who are best positioned to determine when and how interventions should be triggered.

This study has limitations regarding the nature of the data and the model. We used surveillance data, which is known to be prone to under-reporting. Under-reporting may vary in space-time, which hinders the ability of a model to estimate it. Laboratory confirmation is low among our study population, and other co-circulating diseases with similar clinical symptoms could be reported as dengue cases. As mentioned, dengue serotype circulation is an important factor influencing dengue epidemics. However, such information is limited, lacking enough spatio-temporal resolution, and is not publicly available in Brazil. Finally, our model does not account for population- and ecological-level factors that can contribute to dengue transmission and distribution. However, models based only on case data have the advantage of being more straightforward to implement and apply in different contexts. In a forecasting challenge for predicting neuroinvasive disease caused by West Nile virus, another mosquito-borne disease, simple models based on historical cases generally performed better than more complex models (Holcomb et al., 2023).

## 5 Conclusions

Brazil has continental dimensions and a large proportion of dengue control actions are coordinated at the national level. Therefore, identifying the heterogeneity in the magnitude and timing of the epidemic scenarios, both between and within Federal Units, is essential for better public health preparedness and response. Forecasting tools can assist in planning interventions, making them more precisely focused in both time and space. The fact that our forecasting model only needs case data makes it widely applicable. Both our model and the proposed classification of probabilistic epidemic bands can be applied in different countries and for other diseases, provided it is endemic and systematic surveillance data are available over a sufficient period. We recommend using at least five years of data and conducting out-of-sample validation to effectively assess the model's performance.

## CRediT authorship contribution statement

**Laís Picinini Freitas:** Writing – review & editing, Methodology, Conceptualization, Writing – original draft, Funding acquisition. **Danielle Andreza da Cruz Ferreira:** Writing – review & editing, Writing – original draft. **Raquel Martins Lana:** Writing – review & editing, Writing – original draft. **Daniel Cardoso Portela Câmara:** Writing – review & editing, Writing – original draft. **Tatiana P. Portella:** Writing – original draft, Writing – review & editing. **Marilia Sá Carvalho:** Writing – review & editing, Conceptualization, Funding acquisition. **Ayrton Sena Gouveia:** Writing – review & editing. **Iasmim Ferreira de Almeida:** Writing – review & editing. **Eduardo Correa Araujo:** Writing – review & editing. **Luã Bida Vacaro:** Writing – review & editing. **Fabiana Ganem:** Writing – review & editing. **Oswaldo Gonçalves Cruz:** Conceptualization, Writing – review & editing. **Flávio Codeço Coelho:** Conceptualization, Writing – review & editing. **Claudia Torres Codeço:** Conceptualization, Writing – review & editing. **Luiz Max Carvalho:** Writing – review & editing, Methodology, Writing – original draft. **Leonardo Soares Bastos:** Writing – original draft, Funding acquisition, Data curation, Writing – review & editing, Project administration, Formal analysis, Conceptualization.

## Data availability statement

The data underlying this study are publicly available at https://github.com/AlertaDengue/baseline_paper/.

## Funding

10.13039/100020229LPF and LSB are supported by a grant from the 10.13039/100012416Inova/Fiocruz/10.13039/501100006507Oswaldo Cruz Foundation, Brazil and the Department of Public Health Emergencies of the Secretariat for Health and Environmental Surveillance of the Ministry of Health (DEMSP/SVSA/MS) - Brazil [VPPCB-002-FIO-20-2-27], and by the 10.13039/501100003593National Council for Scientific and Technological Development (CNPq), Brazil and the Department of Science and Technology of Secretariat of Science, Technology, Innovation and Health Complex of the Ministry of Health of Brazil (Decit/SECTICS/MS) - Brazil [444896/2023-6]. DACF is supported by 10.13039/501100003593CNPq - Brazil [200453/2024-6]. RML was funded by European Union (Marie Sklodowska-Curie Actions) [grant agreement 101109642]. MSC acknowledges support from 10.13039/501100003593CNPq – Brazil [307450/2021-0] and 10.13039/501100004586Fundação Carlos Chagas Filho de Amparo à Pesquisa do Estado do Rio de Janeiro (FAPERJ) – Brazil [E−26/203.967/2024]. LSB acknowledges support from 10.13039/501100003593CNPq – Brazil [310530/2021-0] and 10.13039/501100004586FAPERJ – Brazil [E−26/201.277/2021 and E−26/204.098/2024].

## Declaration of competing interest

The authors declare that they have no known competing financial interests or personal relationships that could have appeared to influence the work reported in this paper.
