# Supplementary material for: A statistical model for forecasting probabilistic epidemic bands for dengue cases in Brazil
Source: Infect Dis Model. 2025 Aug 5;10(4):1479–87. doi: 10.1016/j.idm.2025.07.014 (PMC12359213; doi:10.1016/j.idm.2025.07.014)
Supplement: Multimedia component 1 [file mmc1.pdf]

A statistical model for forecasting probabilistic epidemic bands for dengue cases in Brazil

Laís Picinini Freitas, Danielle Andreza da Cruz Ferreira, Raquel Martins Lana, Daniel Cardoso Portela Câmara, Tatiana P. Portella, Marilia Sá Carvalho, Ayrton Sena Gouveia, Iasmim Ferreira de Almeida, Eduardo Correa Araujo, Luã Bida Vacaro, Fabiana Ganem, Oswaldo Gonçalves Cruz, Flávio Codeço Coelho, Claudia Torres Codeço, Luiz Max Carvalho, Leonardo Soares Bastos.

Appendix A. Supplementary Figures

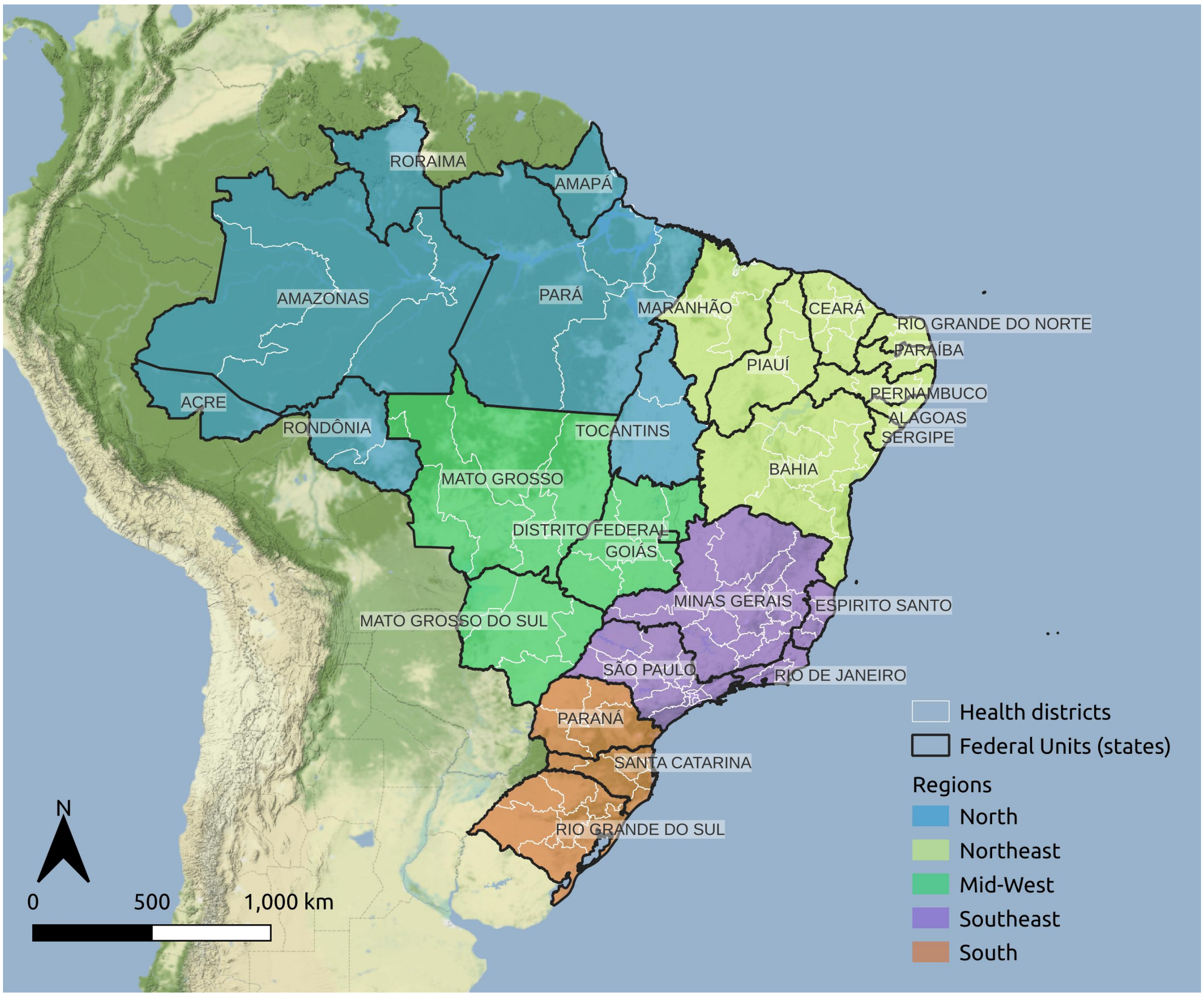

Figure A.1: Divisions of Brazil's territory, 2020.

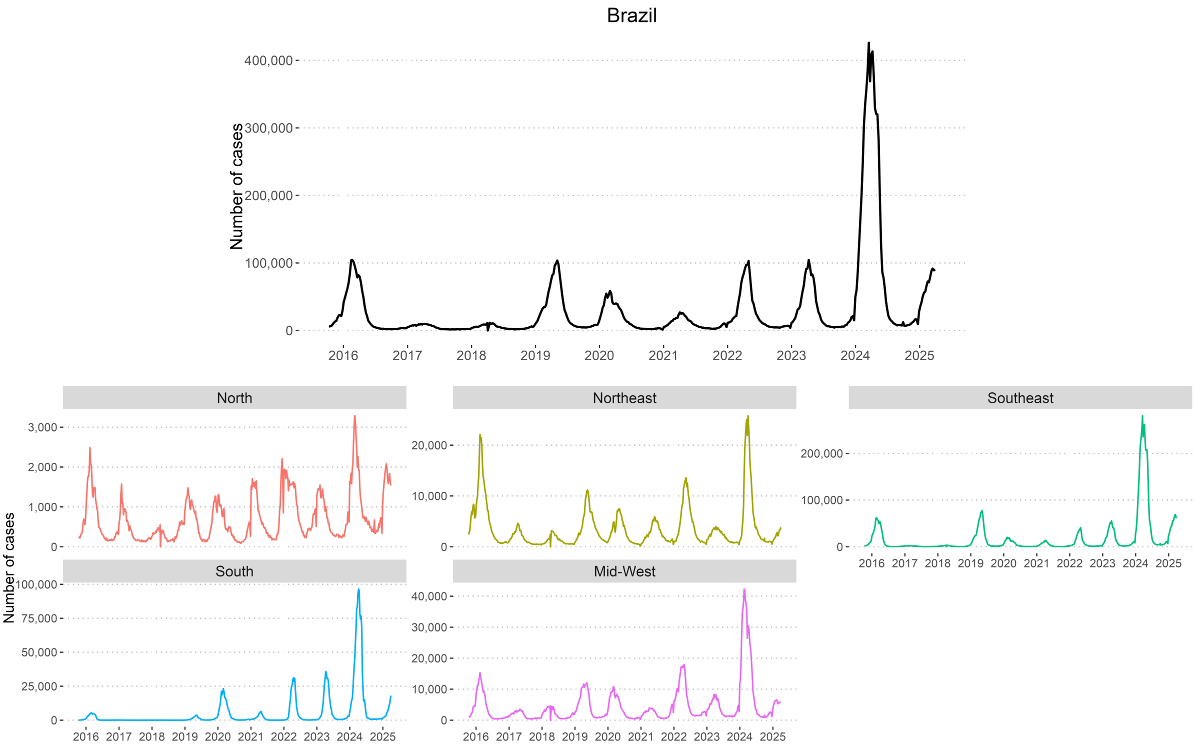

Figure A.2: Weekly number of probable dengue cases in Brazil and by region, 2015 to March 2025.

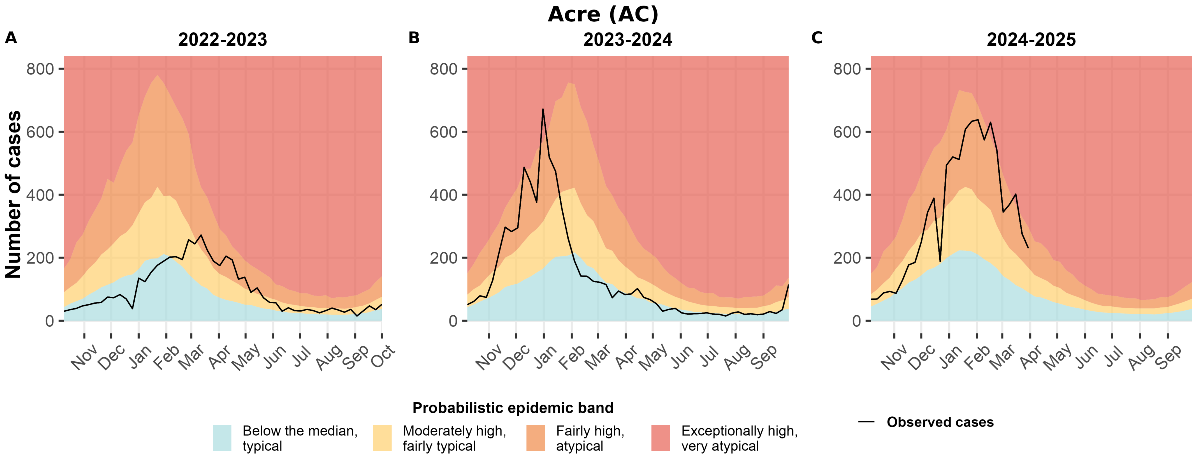

Figure A.3: Estimated probabilistic epidemic bands compared with the observed number of dengue cases by week for seasons (A) 2022-2023, B) 2023-2024, and C) 2024-2025, Acre (AC), Brazil.

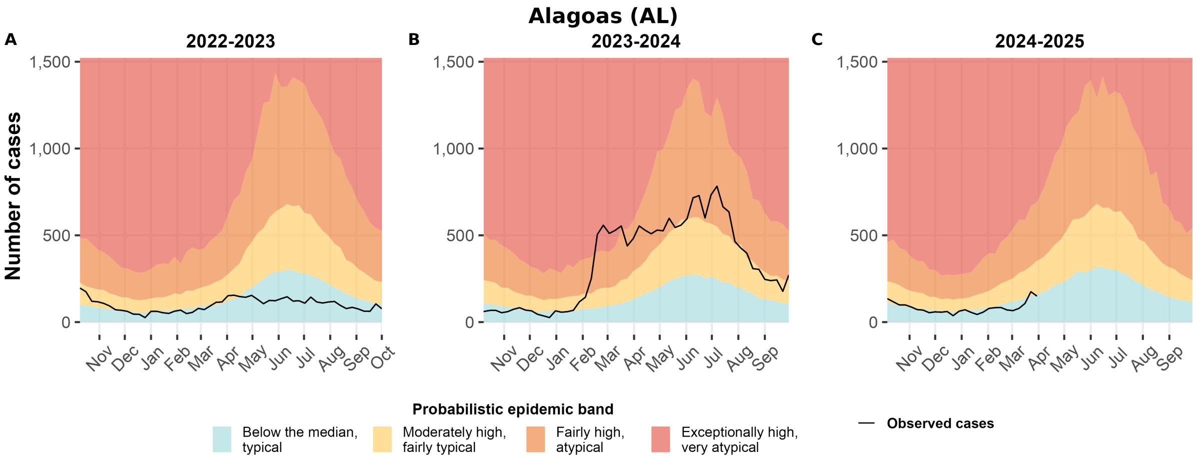

Figure A.4: Estimated probabilistic epidemic bands compared with the observed number of dengue cases by week for seasons (A) 2022-2023, B) 2023-2024, and C) 2024-2025, Alagoas (AL), Brazil.

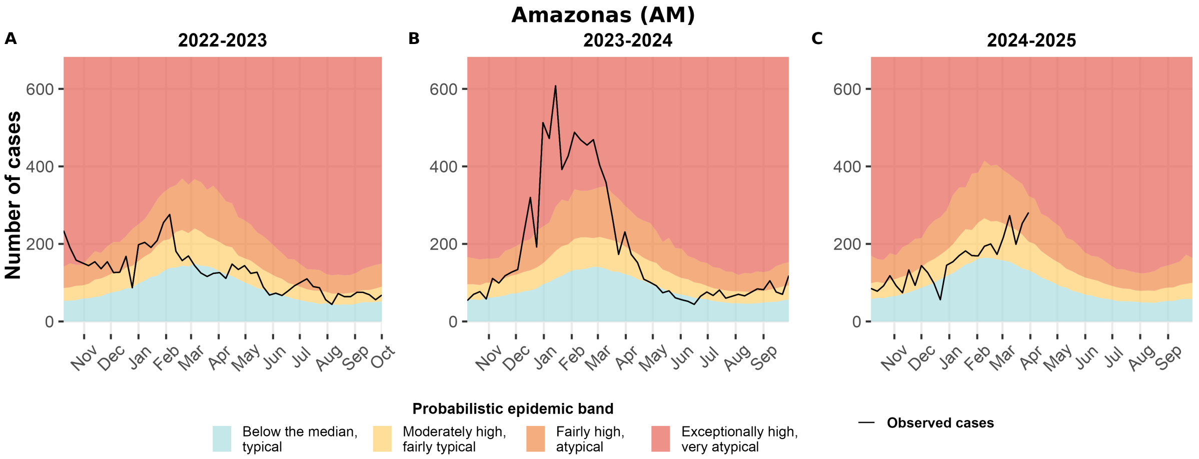

Figure A.5: Estimated probabilistic epidemic bands compared with the observed number of dengue cases by week for seasons (A) 2022-2023, B) 2023-2024, and C) 2024-2025, Amazonas (AM), Brazil.

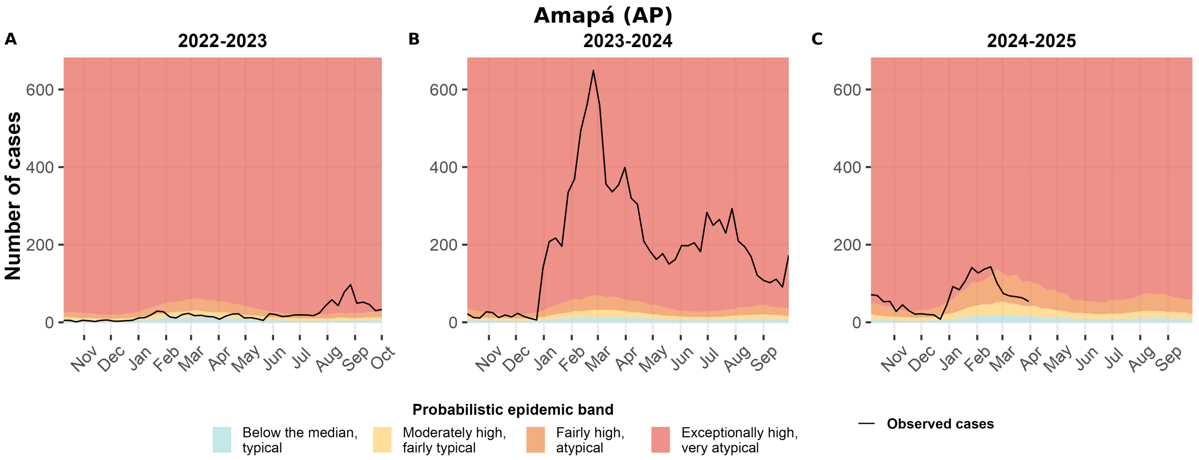

Figure A.6: Estimated probabilistic epidemic bands compared with the observed number of dengue cases by week for seasons (A) 2022-2023, B) 2023-2024, and C) 2024-2025, Amapá (AP), Brazil.

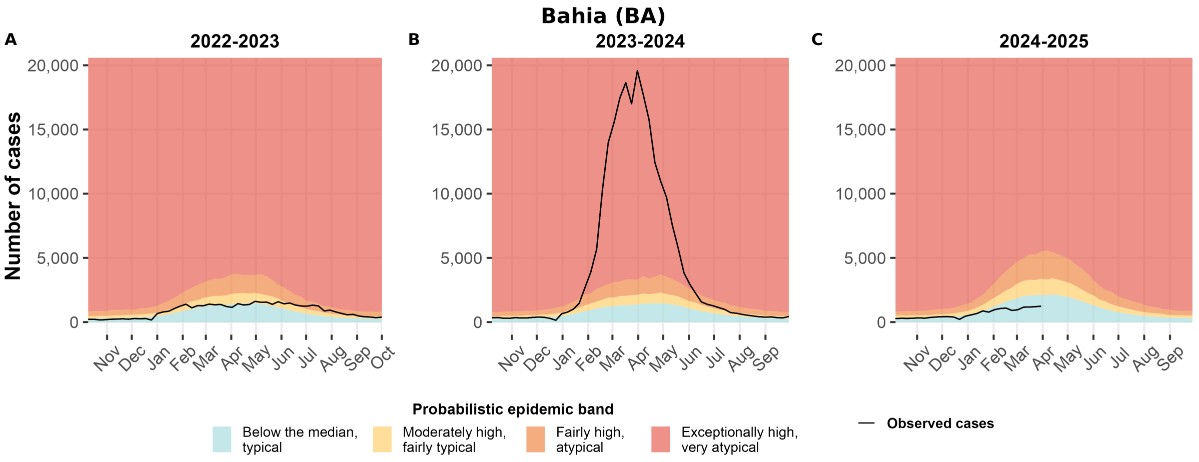

Figure A.7: Estimated probabilistic epidemic bands compared with the observed number of dengue cases by week for seasons (A) 2022-2023, B) 2023-2024, and C) 2024-2025, Bahia (BA), Brazil.

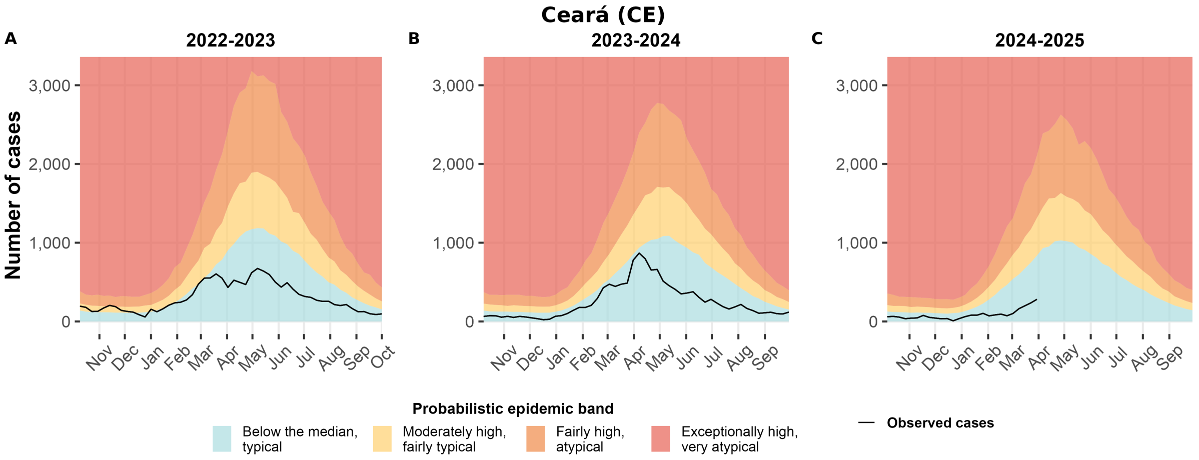

Figure A.8: Estimated probabilistic epidemic bands compared with the observed number of dengue cases by week for seasons (A) 2022-2023, B) 2023-2024, and C) 2024-2025, Ceará (CE), Brazil.

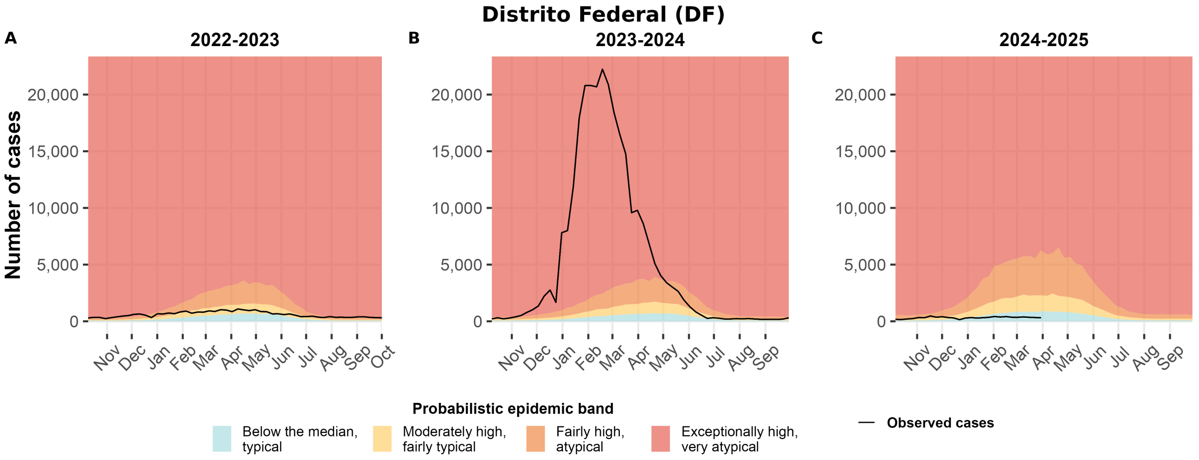

Figure A.9: Estimated probabilistic epidemic bands compared with the observed number of dengue cases by week for seasons (A) 2022-2023, B) 2023-2024, and C) 2024-2025, Distrito Federal (DF), Brazil.

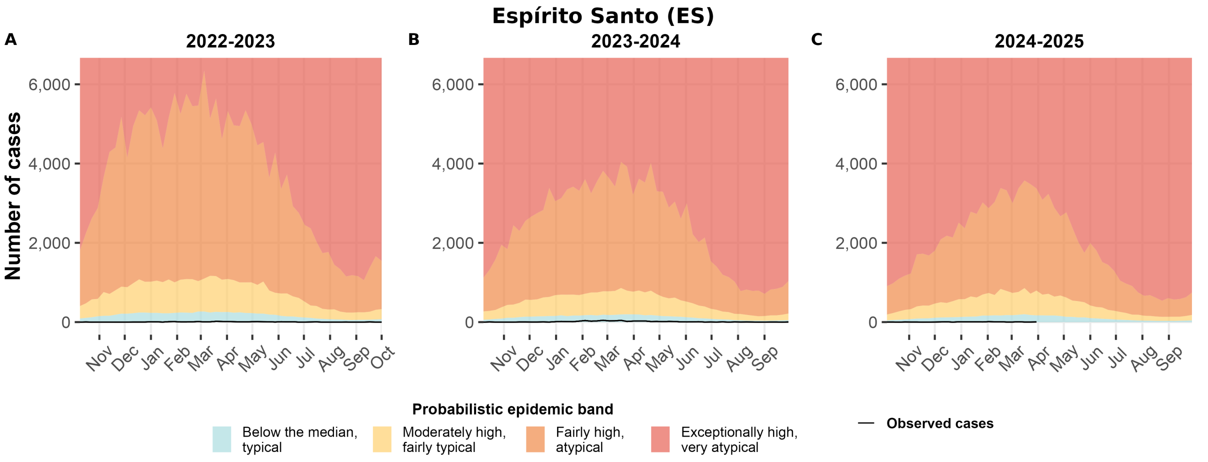

Figure A.10: Estimated probabilistic epidemic bands compared with the observed number of dengue cases by week for seasons (A) 2022-2023, B) 2023-2024, and C) 2024-2025, Espírito Santo (ES), Brazil.

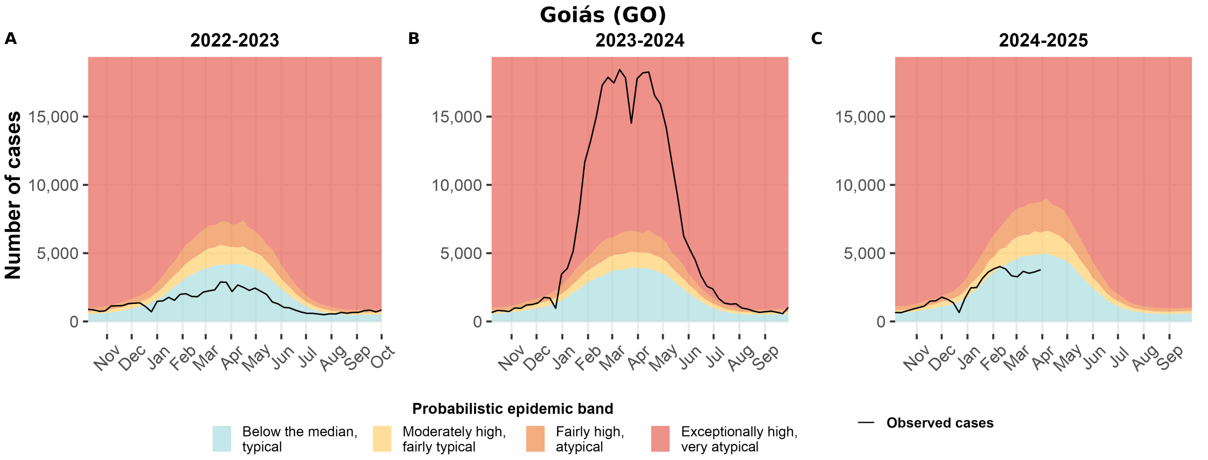

Figure A.11: Estimated probabilistic epidemic bands compared with the observed number of dengue cases by week for seasons (A) 2022-2023, B) 2023-2024, and C) 2024-2025, Goiás (GO), Brazil.

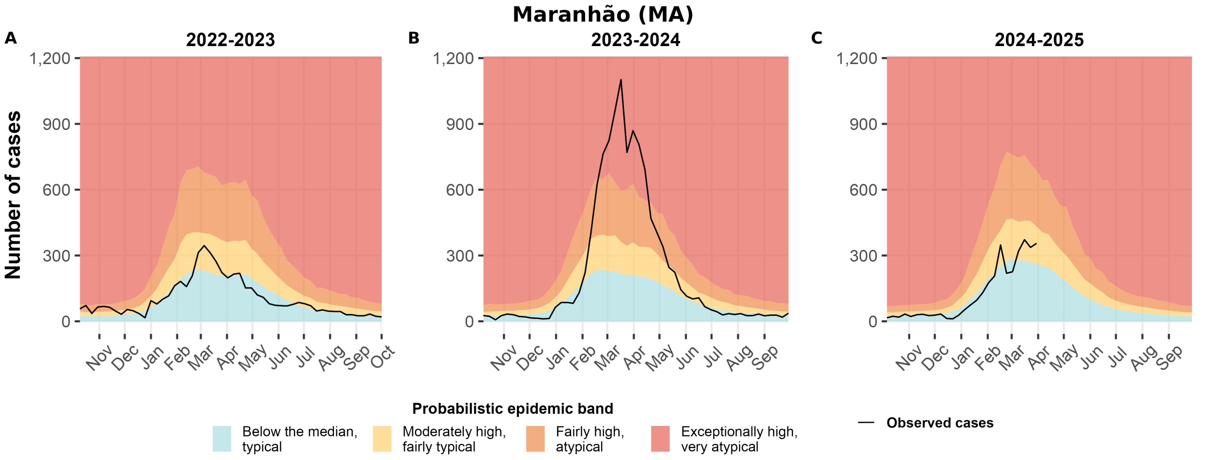

Figure A.12: Estimated probabilistic epidemic bands compared with the observed number of dengue cases by week for seasons (A) 2022-2023, B) 2023-2024, and C) 2024-2025, Maranhão (MA), Brazil.

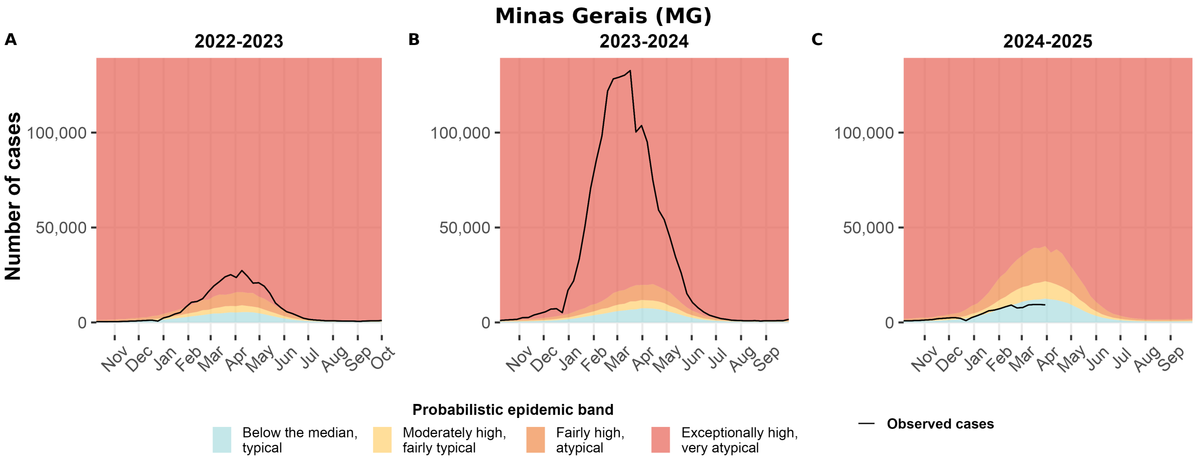

Figure A.13: Estimated probabilistic epidemic bands compared with the observed number of dengue cases by week for seasons (A) 2022-2023, B) 2023-2024, and C) 2024-2025, Minas Gerais (MG), Brazil.

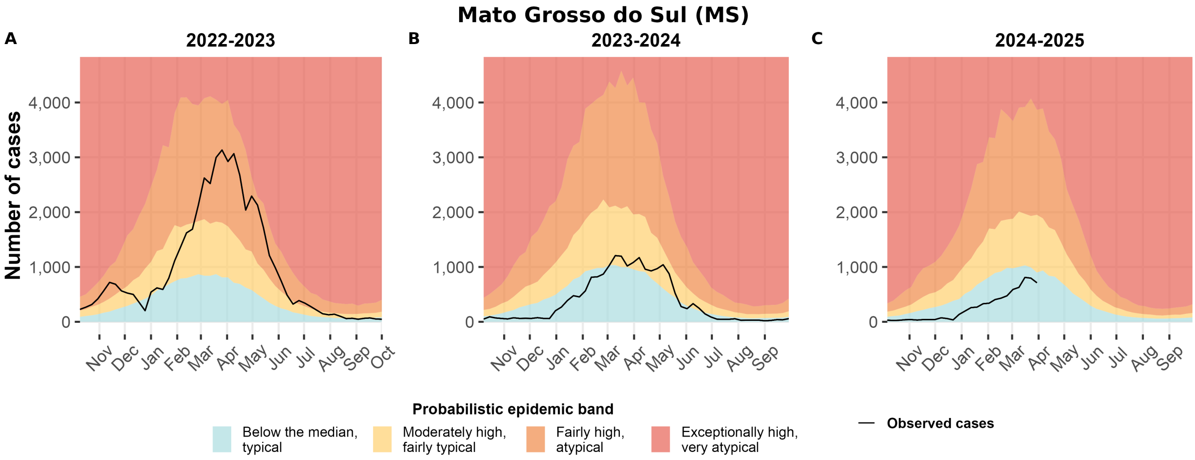

Figure A.14: Estimated probabilistic epidemic bands compared with the observed number of dengue cases by week for seasons (A) 2022-2023, B) 2023-2024, and C) 2024-2025, Mato Grosso do Sul (MS), Brazil.

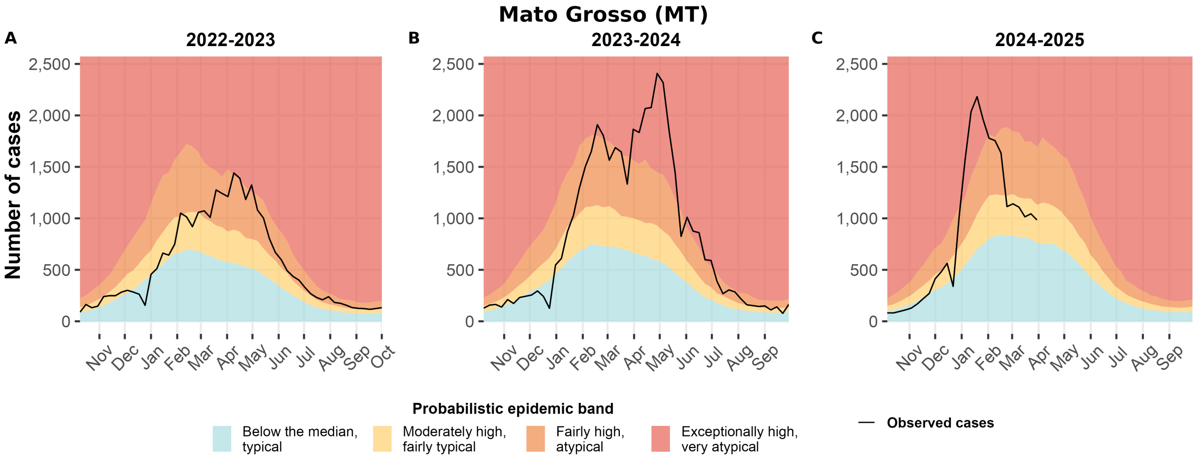

Figure A.15: Estimated probabilistic epidemic bands compared with the observed number of dengue cases by week for seasons (A) 2022-2023, B) 2023-2024, and C) 2024-2025, Mato Grosso (MT), Brazil.

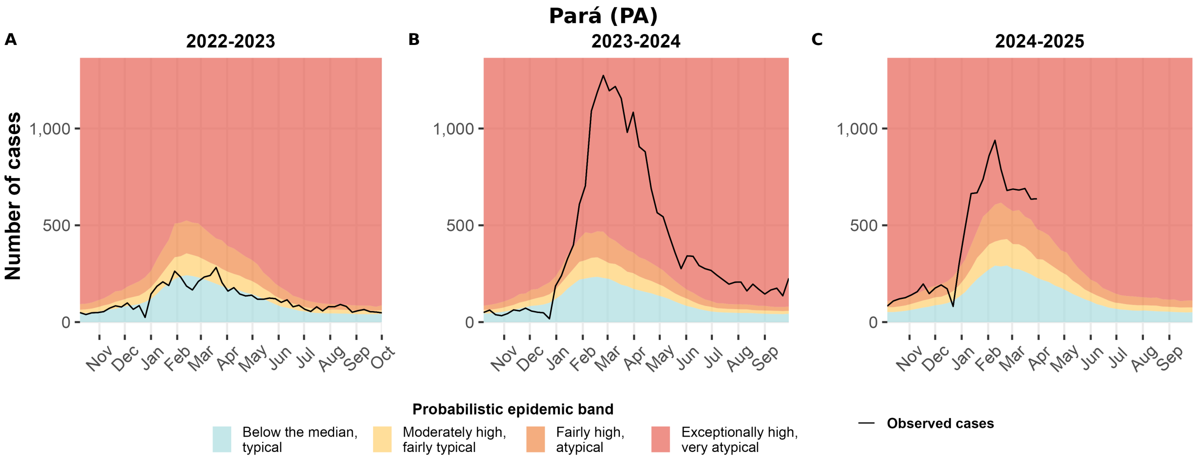

Figure A.16: Estimated probabilistic epidemic bands compared with the observed number of dengue cases by week for seasons (A) 2022-2023, B) 2023-2024, and C) 2024-2025, Pará (PA), Brazil.

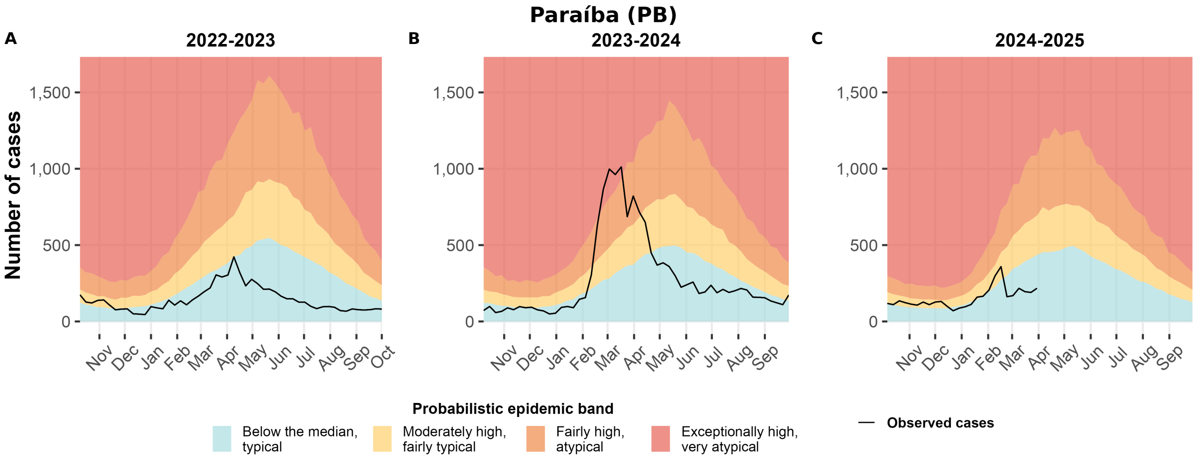

Figure A.17: Estimated probabilistic epidemic bands compared with the observed number of dengue cases by week for seasons (A) 2022-2023, B) 2023-2024, and C) 2024-2025, Paraíba (PB), Brazil.

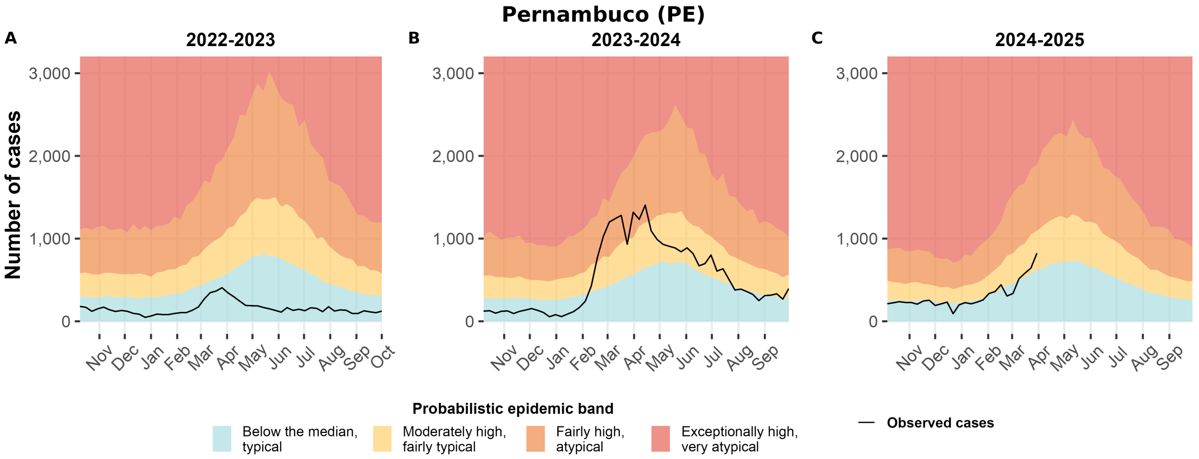

Figure A.18: Estimated probabilistic epidemic bands compared with the observed number of dengue cases by week for seasons (A) 2022-2023, B) 2023-2024, and C) 2024-2025, Pernambuco (PE), Brazil.

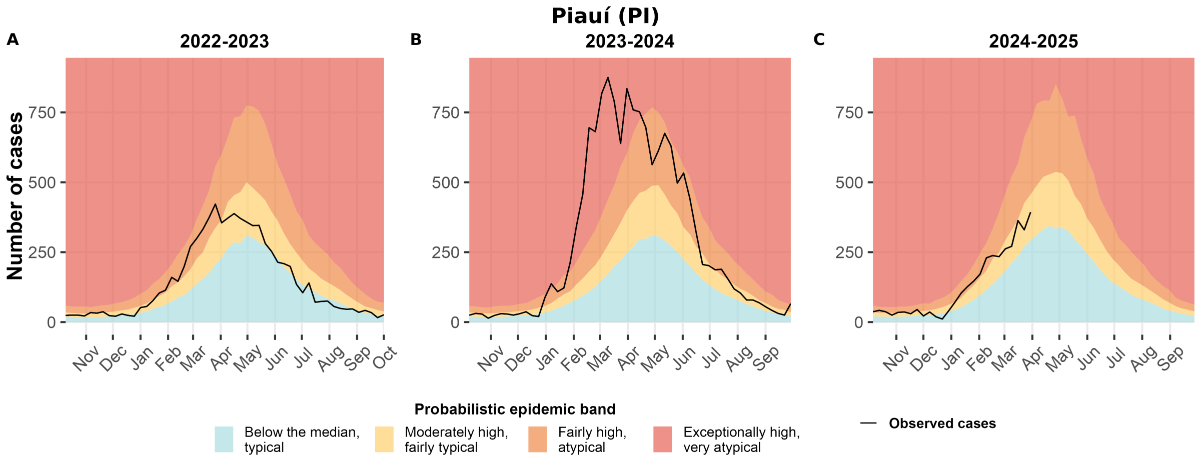

Figure A.19: Estimated probabilistic epidemic bands compared with the observed number of dengue cases by week for seasons (A) 2022-2023, B) 2023-2024, and C) 2024-2025, Piauí (PI), Brazil.

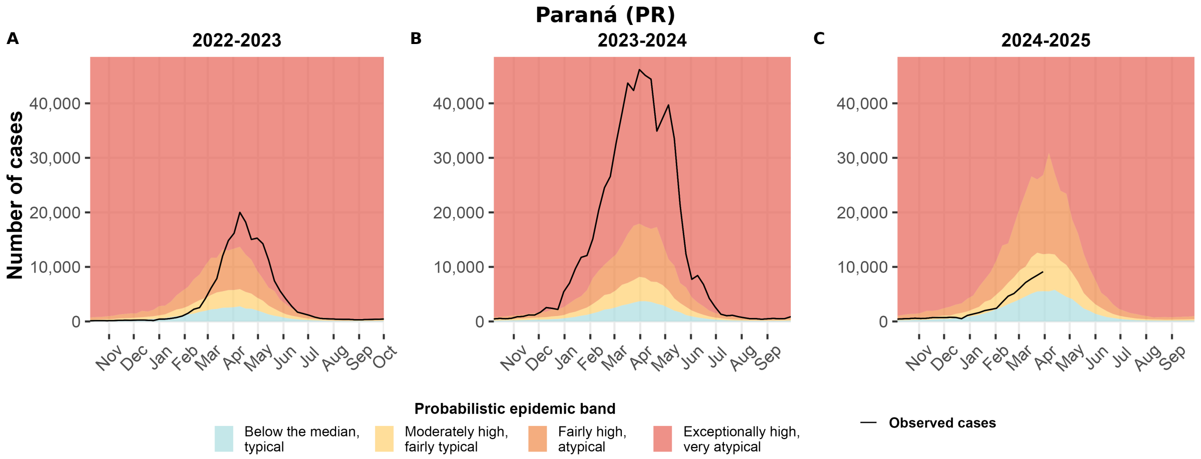

Figure A.20: Estimated probabilistic epidemic bands compared with the observed number of dengue cases by week for seasons (A) 2022-2023, B) 2023-2024, and C) 2024-2025, Paraná (PR), Brazil.

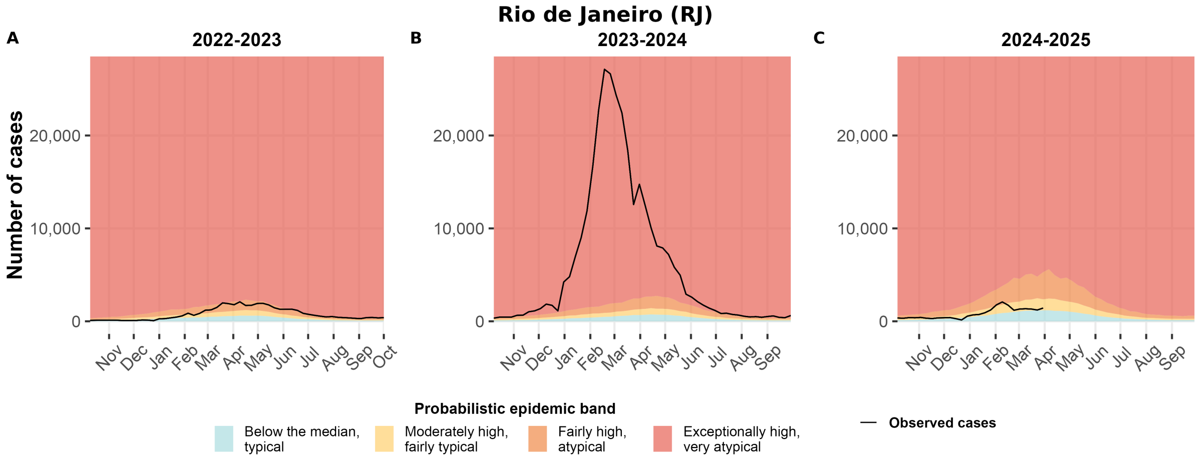

Figure A.21: Estimated probabilistic epidemic bands compared with the observed number of dengue cases by week for seasons (A) 2022-2023, B) 2023-2024, and C) 2024-2025, Rio de Janeiro state (RJ), Brazil.

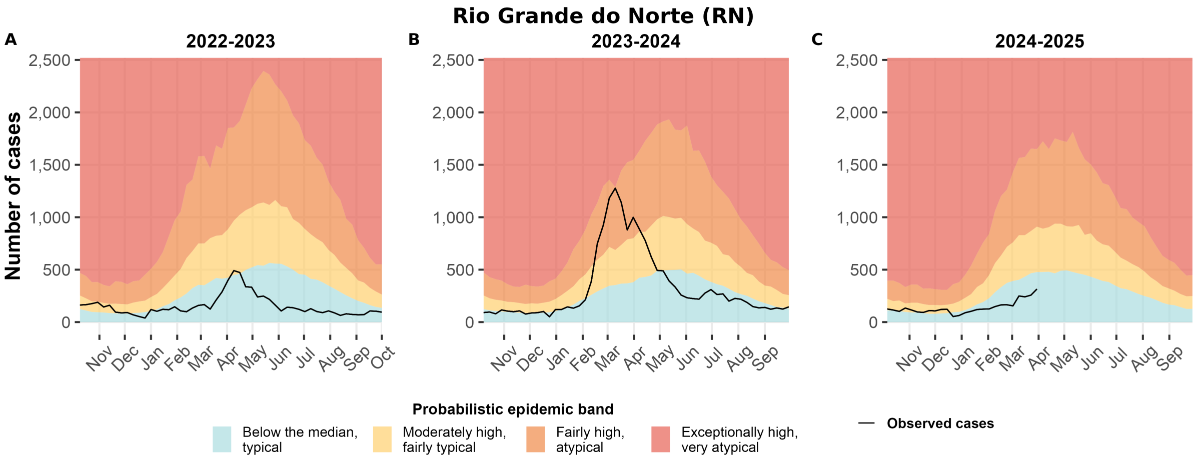

Figure A.22: Estimated probabilistic epidemic bands compared with the observed number of dengue cases by week for seasons (A) 2022-2023, B) 2023-2024, and C) 2024-2025, Rio Grande do Norte (RN), Brazil.

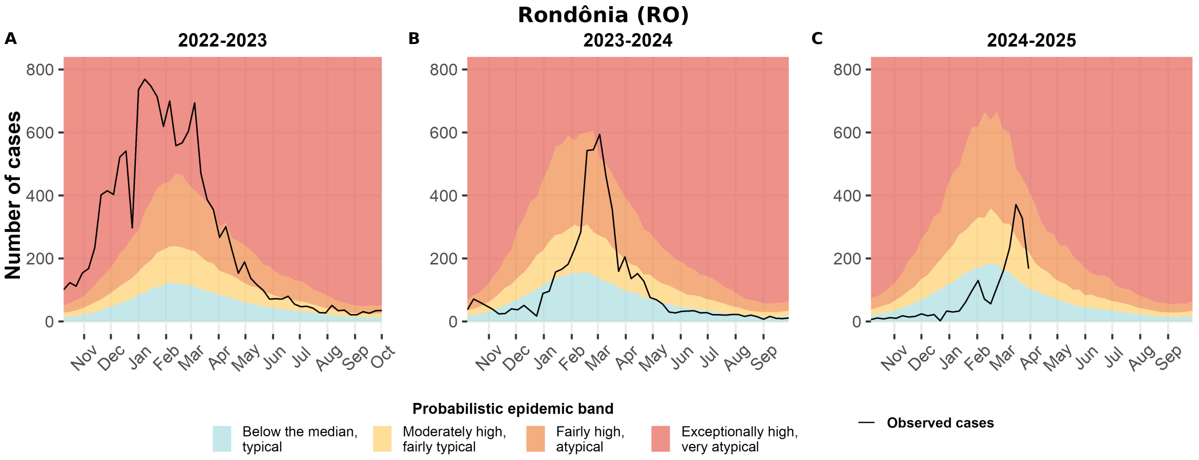

Figure A.23: Estimated probabilistic epidemic bands compared with the observed number of dengue cases by week for seasons (A) 2022-2023, B) 2023-2024, and C) 2024-2025, Rondônia (RO), Brazil.

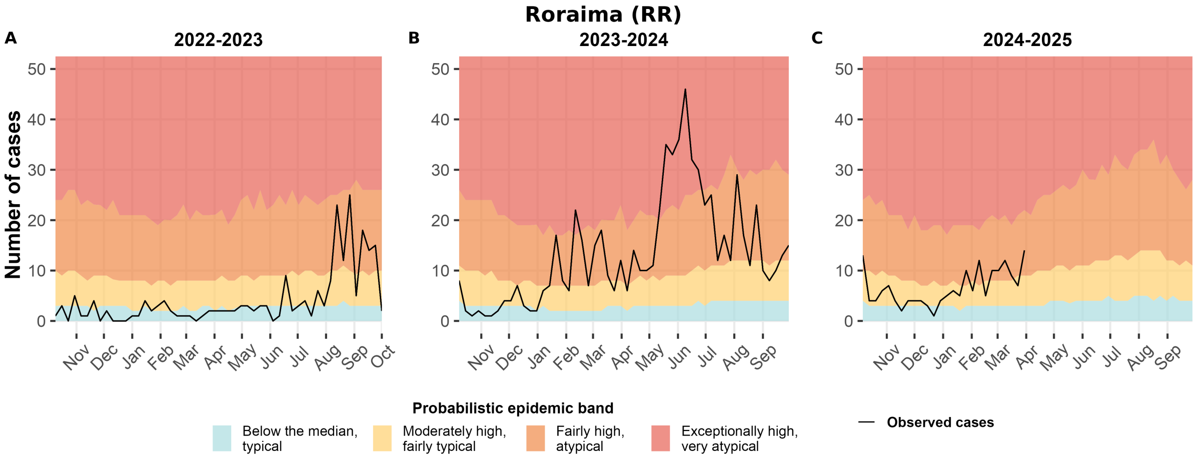

Figure A.24: Estimated probabilistic epidemic bands compared with the observed number of dengue cases by week for seasons (A) 2022-2023, B) 2023-2024, and C) 2024-2025, Roraima (RR), Brazil.

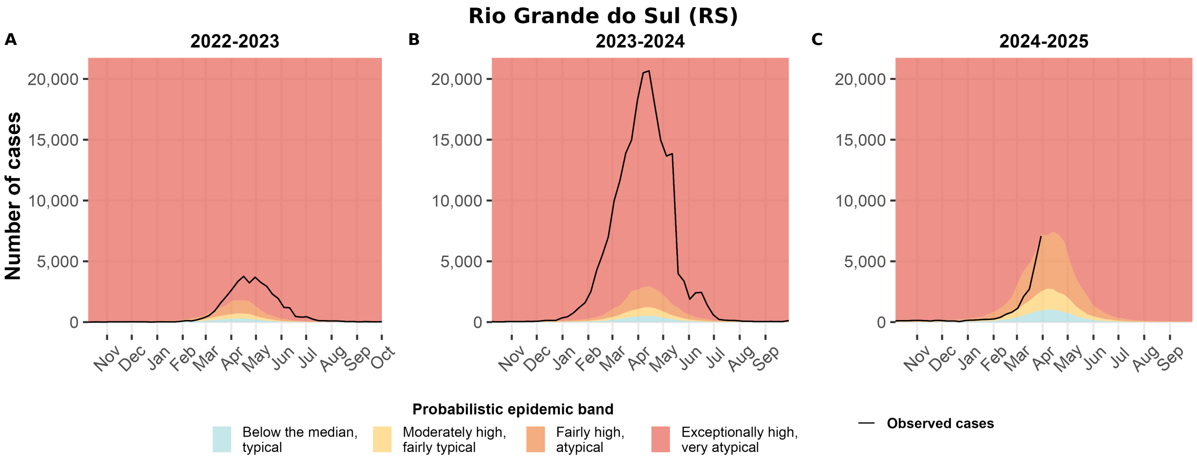

Figure A.25: Estimated probabilistic epidemic bands compared with the observed number of dengue cases by week for seasons (A) 2022-2023, B) 2023-2024, and C) 2024-2025, Rio Grande do Sul (RS), Brazil.

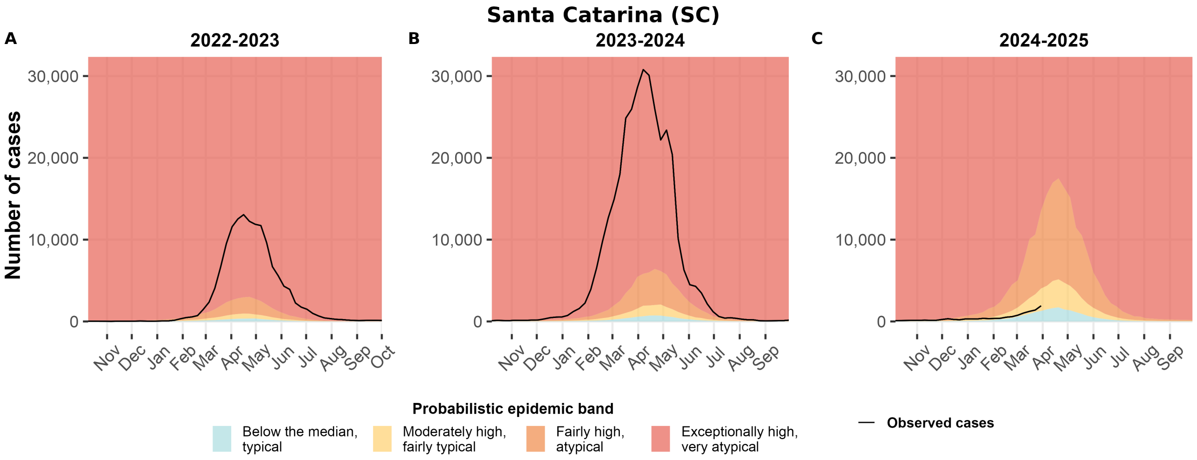

Figure A.26: Estimated probabilistic epidemic bands compared with the observed number of dengue cases by week for seasons (A) 2022-2023, B) 2023-2024, and C) 2024-2025, Santa Catarina (SC), Brazil.

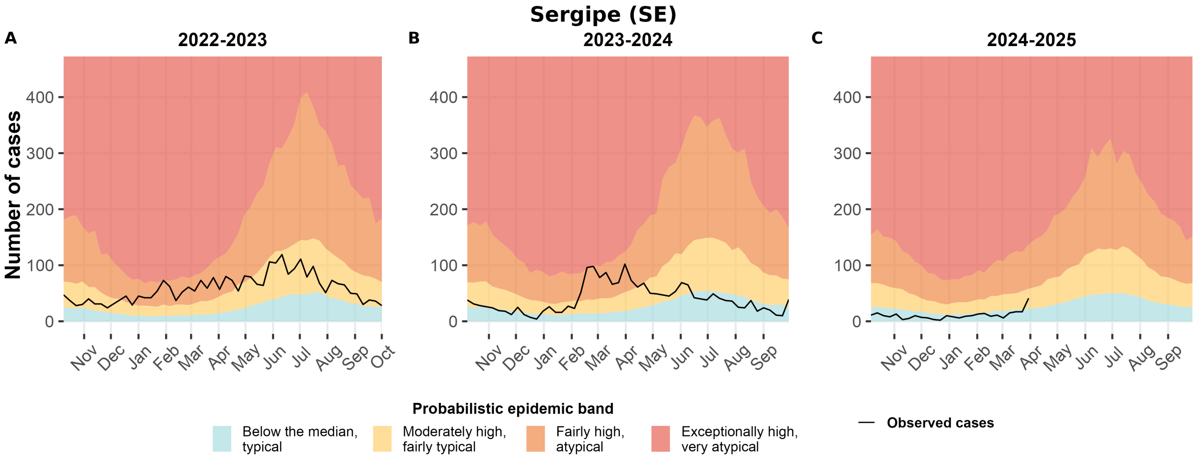

Figure A.27: Estimated probabilistic epidemic bands compared with the observed number of dengue cases by week for seasons (A) 2022-2023, B) 2023-2024, and C) 2024-2025, Sergipe (SE), Brazil.

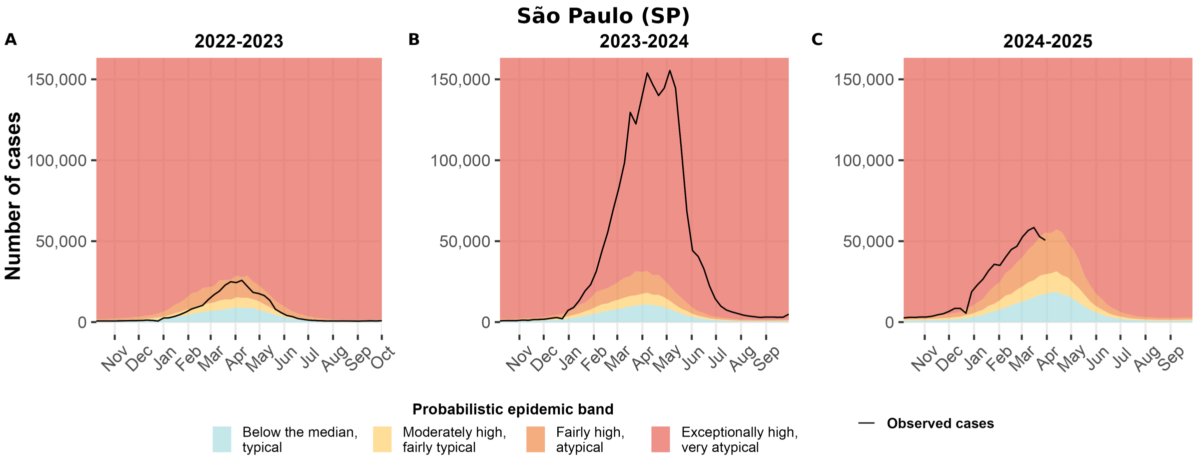

Figure A.28: Estimated probabilistic epidemic bands compared with the observed number of dengue cases by week for seasons (A) 2022-2023, B) 2023-2024, and C) 2024-2025, São Paulo state (SP), Brazil.

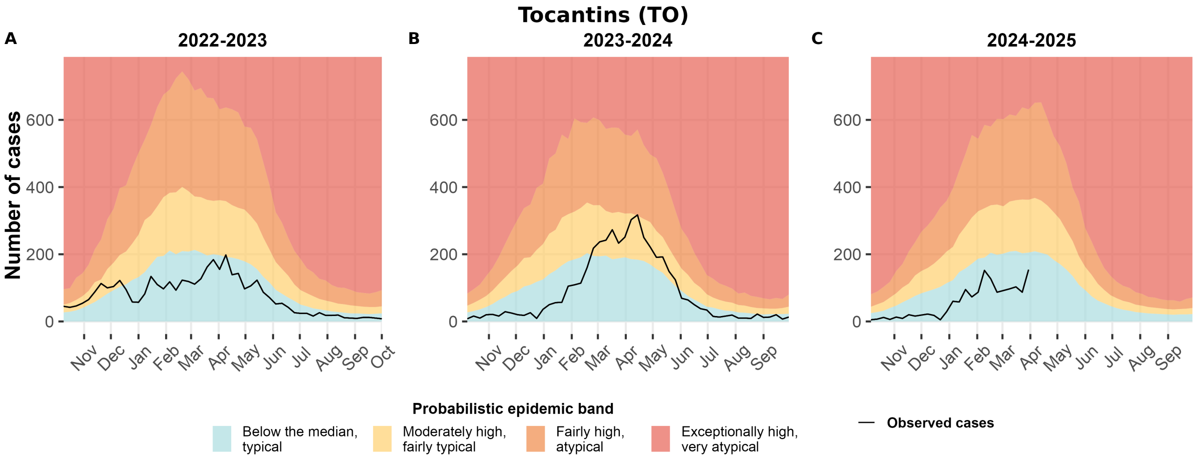

Figure A.29: Estimated probabilistic epidemic bands compared with the observed number of dengue cases by week for seasons (A) 2022-2023, B) 2023-2024, and C) 2024-2025, Tocantins (TO), Brazil.

Appendix B. Comparative analysis with control charts

We conducted a comparative analysis between our model's probabilistic epidemic bands and the control charts recommended by the Brazilian Ministry of Health for outbreak monitoring (Brasil, Ministério da Saúde et al., 2025). The Ministry's control charts are constructed using historical data from the previous five years, based on the median, first quartile (Q1), and third quartile (Q3) of weekly dengue case counts, thereby forming an endemic channel to identify abnormal epidemic activity.

For the purpose of this analysis only, we considered the Q3 threshold (75th percentile) as the criterion for issuing a warning. In our probabilistic model, exceeding the "moderately high, fairly typical" band approximately corresponds to crossing this Q3 threshold. We applied this comparison across three dengue seasons: 2022–2023, 2023–2024, and 2024–2025 (Figure B.30.

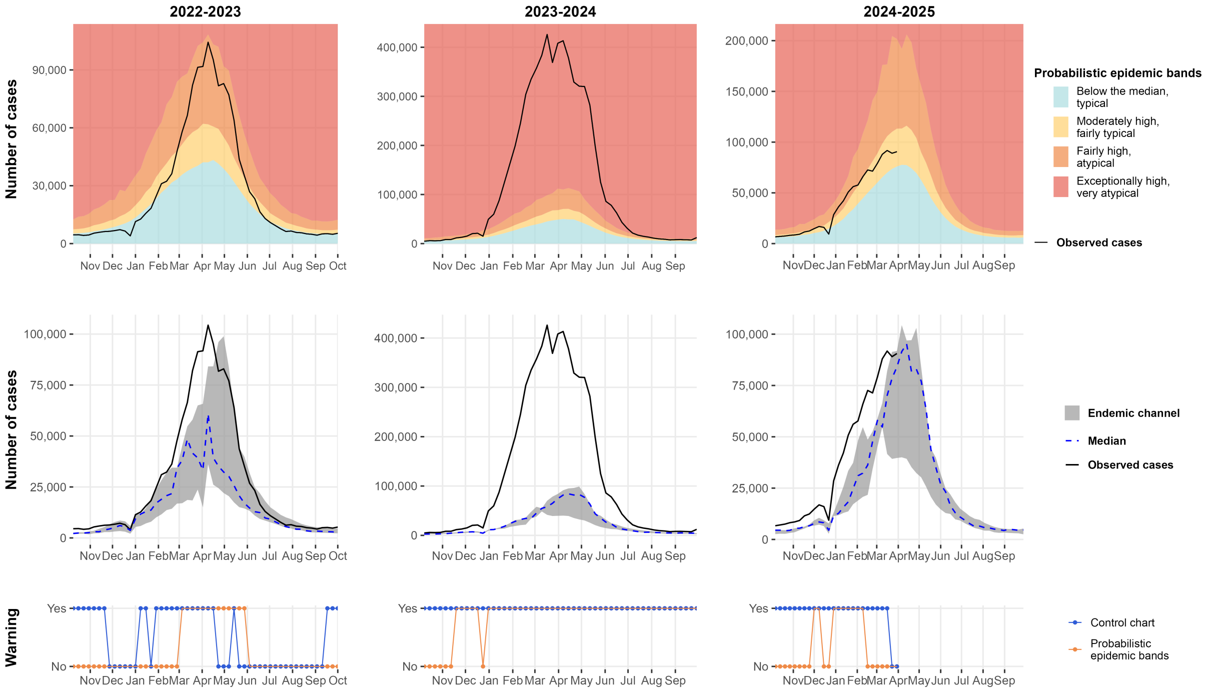

Figure B.30: Estimated probabilistic epidemic bands compared with the observed number of dengue cases by week for seasons (A) 2022-2023, B) 2023-2024, and C) 2024-2025, Tocantins (TO), Brazil.

It is important to note that interpretation of this comparison must be approached with caution, as there is no established gold standard for outbreak warnings.

Overall, our analysis revealed that the Ministry's control charts tended to generate more frequent warnings, including during periods of low case counts and without clear upward trends, such as observed early in the 2022–2023 season. The control charts also exhibited greater sensitivity to temporal shifts in epidemic patterns, triggering alerts when outbreaks shifted in timing but not necessarily in magnitude, as seen in the 2024–2025 season.

Conversely, the probabilistic epidemic bands issued fewer alerts, which appeared to be more robust and accurate. These bands demonstrated reduced sensitivity to minor fluctuations and better reflected substantial departures from expected epidemic behaviour.

These findings illustrate the trade-off between sensitivity and specificity inherent in outbreak alert systems. Our probabilistic approach may reduce false alarms caused by

minor temporal variations or small increases in case counts, thus potentially providing more reliable support for public health decision-making in real time.

References

Brasil, Ministério da Saúde, Secretaria de Vigilância em Saúde e Ambiente, Departamento de Doenças Transmissíveis, 2025. Plano de contingência nacional para dengue, chikungunya e Zika. URL: http://bvsms.saude.gov.br/bvs/publicacoes/plano_contingencia_nacional_dengue_zika.pdf. brasília : Ministério da Saúde, 102 p. : il. ISBN 978-65-5993-709-7.
